# Spatial pattern of plutonium and radiocaesium contamination released during the Fukushima Daiichi nuclear power plant disaster

**DOI:** 10.1038/s41598-018-34302-0

**Published:** 2018-11-14

**Authors:** James A. Dunne, Peter G. Martin, Yosuke Yamashiki, Ian X. Y. Ang, Tom B. Scott, David A. Richards

**Affiliations:** 1 School of Geographical Sciences, University of Bristol, University Road, Bristol, BS8 1SS UK; 2 Bristol Isotope Group, University of Bristol, Wills Memorial Building, Queen’s Road, Bristol, BS8 1RJ UK; 3 Interface Analysis Centre, HH Wills Physics Laboratory, University of Bristol, Bristol, BS8 1TL UK; 4 Graduate School of Advanced Integrated Studies in Human Survivability, Kyoto University, Kyoto, 606–8501 Japan

## Abstract

Plutonium and radiocaesium are hazardous contaminants released by the Fukushima Daiichi nuclear power plant (FDNPP) disaster and their distribution in the environment requires careful characterisation using isotopic information. Comprehensive spatial survey of ^134^Cs and ^137^Cs has been conducted on a regular basis since the accident, but the dataset for ^135^Cs/^137^Cs atom ratios and trace isotopic analysis of Pu remains limited because of analytical challenges. We have developed a combined chemical procedure to separate Pu and Cs for isotopic analysis of environmental samples from contaminated catchments. Ultra-trace analyses reveal a FDNPP Pu signature in environmental samples, some from further afield than previously reported. For two samples, we attribute the dominant source of Pu to Reactor Unit 3. We review the mechanisms responsible for an emergent spatial pattern in ^134,135^Cs/^137^Cs in areas northwest (high ^134^Cs/^137^Cs, low ^135^Cs/^137^Cs) and southwest (low ^134^Cs/^137^Cs, high ^135^Cs/^137^Cs) of FDNPP. Several samples exhibit consistent ^134,135^Cs/^137^Cs values that are significantly different from those deposited on plant specimens collected in previous works. A complex spatial pattern of Pu and Cs isotopic signature is apparent. To confidently attribute the sources of mixed fallout material, future studies must focus on analysis of individual FDNPP-derived particles.

## Introduction

The release of considerable quantities of radioactive material from the Fukushima Daiichi Nuclear Power Plant (FDNPP) accident in March 2011 contaminated large areas of the surrounding terrestrial environment. The dispersion and deposition of radioactive material were strongly influenced by the prevailing weather systems and a complex pattern of intermittent emission from multiple reactor units^1^. The meteorological conditions during emission periods are well defined, but there remains considerable uncertainty about the isotopic composition and release rates of radioactive contaminants from the various potential sources at FDNPP^2^.

Determining the spatial distribution of radionuclides can be used to infer the quantity of material released, its chemical composition and the environmental pathways. Additionally, subsequent temporal variation provides insight into the mechanisms that transfer or retain radionuclides of interest. Monitoring and understanding post-depositional remobilisation of potentially harmful radionuclides in the environment allows the risk they present to be managed more effectively. This information is highly valued, not only for decision-making processes regarding public health, but also the longer-term remediation efforts across the region and any future nuclear incidents that may occur.

Radiocaesium and Pu are recognised as two of the most significant percieved concerns from the disaster. The release of Pu to terrestrial areas of Japan remained unconfirmed for up to one year following the Fukushima disaster^3^. Since then, several studies have provided evidence of its release to the land surface north, northwest and south of FDNPP, up to distances of 120 km from the site^3–7^. It is estimated that only a fraction (~0.002%) of Pu from the total reactor core inventory was introduced to the environment^3^. For the data available, only trace quantities of FDNPP-derived Pu have been measured in environmental materials and mixing of the FDNPP Pu with trace amounts of Pu derived from atmospheric nuclear weapons testing complicates interpretation of the observed isotopic signature^5,8^. Heterogeneity in both isotopic ratio and specific activity of Pu in the vicinity of FDNPP has raised questions about the source and composition of contamination^4,9^. The low volatility of Pu and the incidence of hydrogen explosions suggests Pu may have been distributed in a particulate form. The introduction of particulate Pu to the environment raises concern because of the risk such material poses to public health^10,11^.

In contrast to Pu, Cs has high volatility. Consequently, an extremely large quantity of radiocaesium was released from FDNPP (9–37 PBq ^137^Cs) and is considered to be the principal source of radiation dose to the public^2,12–14^. Following the FDNPP accident, an intensive programme of ^134^Cs and ^137^Cs measurement was conducted to determine the spatial distribution of these isotopes^15^. Recently, ^134^Cs/^137^Cs data obtained since the accident has been combined with atmospheric transport models to identify the reactor units responsible for the highest dose rate areas^16^. The determination of ^134^Cs/^137^Cs activity ratio has been a useful tool within decontamination efforts, risk assessment and source term attribution following the FDNPP accident, but it is recognised that the additional measurement of ^135^Cs/^137^Cs atom ratio enables much better discrimination of the source terms^6,17–21^. While variations in ^134^Cs/^137^Cs activity ratio are observed due to changes in fuel burnup, ^135^Cs/^137^Cs atom ratios vary as a function of neutron capture by the fission product ^135^Xe, the parent nuclide of ^135^Cs. Concentrations of ^135^Xe change considerably according to reactor conditions^22^. Adding ^135^Cs to the suite of isotopic analyses is necessary because all three reactors were boiling-water reactors with similar fuels (Reactor Units 1 & 2 - UO_2_; Reactor Unit 3 - UO_2_/MOX) and operating conditions^23^. Also, mass-spectrometric determination of ^135^Cs/^137^Cs can achieve greater relative precisions^20^ and this signal will remain useful in environmental samples for much longer (^134^Cs t_1/2_ = 2.07 a, ^135^Cs t_1/2_ = 2.3 Ma, ^137^Cs t_1/2_ = 30.08 a).

Here, we explore the spatial pattern of the Pu and Cs isotopes in a variety of environmental samples to evaluate the source term, potential environmental pathways and influence of source mixing between FDNPP contamination and atmospheric fallout^9,19,24,25^. Critical, was the development of a new simultaneous procedure to separate both Cs and Pu fractions from the same environmental samples. Isotopic analyses were performed by thermal ionization mass spectrometry (TIMS) and multicollector inductively coupled plasma mass spectrometry (MC-ICPMS). We present ^239+240^Pu activities, ^240^Pu/^239^Pu atom ratios, ^137^Cs activity, ^134^Cs/^137^Cs activity ratios and ^135^Cs/^137^Cs atom ratios for samples collected from locations primarily in the Fukushima prefecture.

## Results

**Figure 1:**
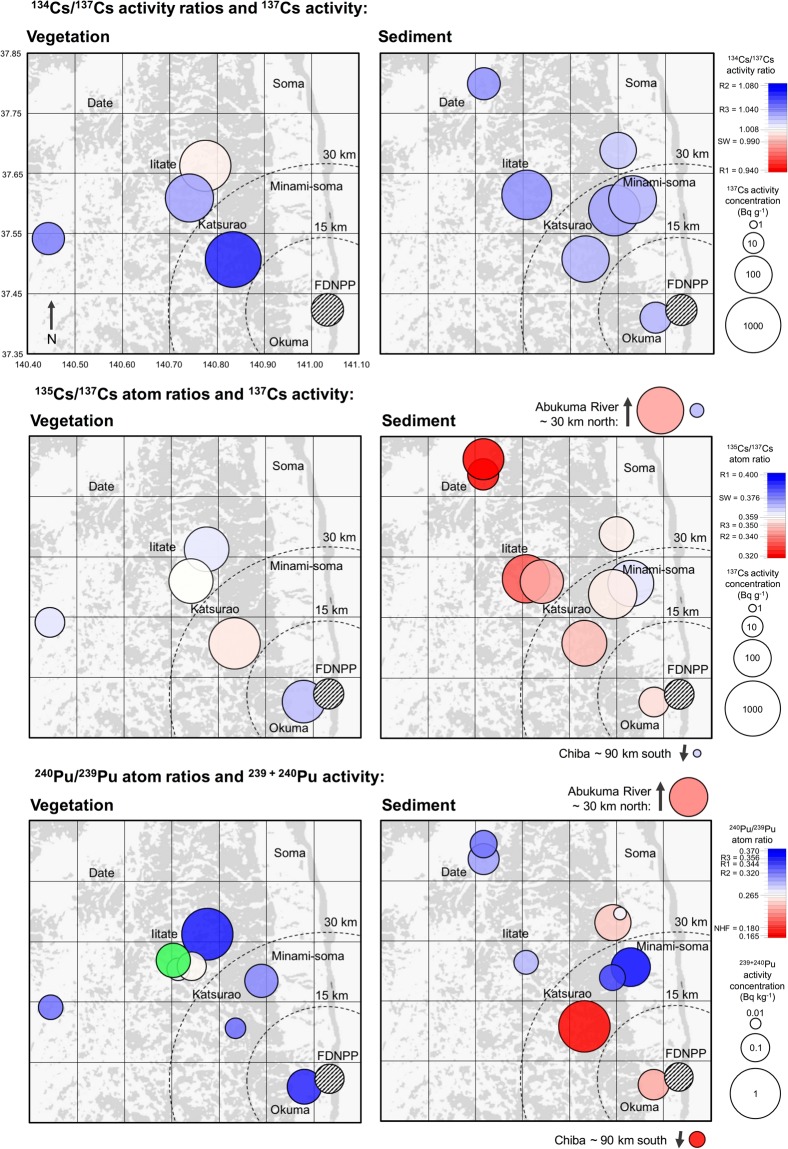
Isoscapes of ^134, 135, 137^Cs and ^239, 240^Pu for part of the Fukushima prefecture surrounding FDNPP. The green marker is used to highlight an anomalous ^240^Pu/^239^Pu atom ratio of 0.64. R1, R2 and R3 correspond to ORIGEN estimated isotope ratio values for Reactor Units 1, 2 and 3, respectively^27^. SW indicates the mean value for the Cs isotope ratios measured to the southwest of FDNPP by Snow *et al*.^19^. ^240^Pu/^239^Pu atom ratio for Northern Hemisphere integrated global fallout is denoted by NHF^28^.

Isotopic analysis of Pu and Cs has been performed on range of environmental samples (soil, sediment and various types of vegetation), collected from several locations in the Fukushima prefecture, two from the Abukuma River and one sample from Chiba, near Tokyo, during May 2014 and October 2015 (Fig. 1; Tables S1 and S2). All environmental samples represent, to varying degrees, a combination of background signal, contamination during the FDNPP accident and also post-depositional migration. These factors complicate the interpretation of the spatial distributions of Pu and Cs. We recognise that the affinity of Pu and Cs may vary considerably between even the most similar types of environmental sample as a function of their respective physico-chemical properties. We broadly categorise sample types into *vegetation* and *sediment* for Fig. 1, but recognise that the two populations will exhibit overlap. Reported values of Pu and Cs activity (symbol size) and activity/atom ratios (symbol colour) are for the strong acid leach (8 M HNO_3_) fraction of ~1 g sub-samples. Therefore, these data are representative of the leachable fraction only and are not necessarily indicative of local radioactivity levels. We expect results from vegetation samples (moss, litter etc) to be biased to the more recent deposition of FDNPP contamination, whereas sediment and soil samples are expected to contain a greater proportion of background signal from the intensive period of atmospheric weapons testing during the 1950s and 1960s. All results presented are decay corrected to 11/03/2011.

Specific activity of ^239+240^Pu ranges from 0.012 ± 0.005 Bq kg^−1^ in coarse sediment from Minamisoma to 0.761 ± 0.182 Bq kg^−1^ for a soil sample collected from Katsurao. A sample from Iitate Village exhibited a high ^239+240^Pu activity of 0.757 ± 0.079 Bq kg^−1^, while the remaining samples were ≤0.2 Bq kg^−1^. These ^239+240^Pu specific activities are similar to baseline values (0.004–1.46 Bq kg^−1^) obtained from samples collected across Japan prior to the Fukushima disaster^26^. All ^240^Pu/^239^Pu atom ratios lie between the integrated Northern Hemisphere global fallout value (0.180 ± 0.014) and the ORIGEN estimated value for Reactor Unit 3 (0.356)^27,28^, except one anomaly (Iitate), 0.645 ± 0.024.

Except for two samples collected from Date City, the data obtained for ^135^Cs/^137^Cs atom ratios and ^134^Cs/^137^Cs activity ratios fall within the range of ORIGEN calculated values for reactor cores of Units 1–3. The ^137^Cs activities in these samples range from 1.2–1040 Bq g^−1^.

## Discussion

Of 22 samples analysed in this study, 15 exhibit a ^240^Pu/^239^Pu atom ratio greater than the upper limit of the values reported for pre-FDNPP soils collected from central-eastern Japan in the 1970s^26^. While this is a higher fraction than observed in other recent studies^3,4,25,29^ direct comparisons should avoided because of differences in experimental protocol, i.e. sample type, locations and chemical treatment (leachable fraction). Some of our data illustrates that FDNPP-derived Pu is found in environmental samples collected considerably further afield than previously reported^4,7^: Two of the furthest study sites from FDNPP, Date and Minamiosawa (>50 km direct distance), exhibit FDNPP-derived ^240^Pu/^239^Pu atom ratios of 0.323 ± 0.021 and 0.324 ± 0.029, respectively. Previous efforts to assess the presence of FDNPP-derived Pu in environmental samples >45 km have been compromised by either limits of detection or by material being derived of Pu predominately sourced from weapons testing fallout^7^. While the ^239+240^Pu activity for all of the samples remains similar or lower than typical global fallout measurements for the region (0.5–1.5 Bq kg^−1^)^30^, such a heterogeneous spatial distribution of ^240^Pu/^239^Pu indicates that particulates have been transported further afield from FDNPP than was previously reported.

There is no apparent relationship between ^239+240^Pu activity and ^240^Pu/^239^Pu atom ratio in Fig. 1. Indeed, the two samples with highest ^239+240^Pu activities exhibit end-member values of ^240^Pu/^239^Pu atom ratios representing weapons testing fallout and FDNNP reactor cores; ^240^Pu/^239^Pu = 0.165 ± 0.003 and ^240^Pu/^239^Pu = 0.359 ± 0.013, respectively. We also find a poor relationship between distance from the FDNPP site and Pu isotopic data. One might expect to observe higher ^239+240^Pu activities and ^240^Pu/^239^Pu atom ratios similar to the ORIGEN estimated values (0.320–0.356) at sites closer to FDNPP, but this is not the case. The non-uniform spatial distribution of Pu isotope data is in agreement with previous work^3,4,25^ and may be a product of one or a combination of two possibilities: (1) contamination during FDNPP hydrogen explosions^4^, which may have ejected fuel particles with an uneven size distribution and variable dispersion pathway to the surrounding environment^31^; (2) non-uniform pattern of background, pre-FDNPP, Pu. For the latter case, because FDNPP Pu concentations are similar to background levels, the observed spatial distribution of Pu isotopes may be caused by different degrees of mixing with a non-uniform pre-FDNPP Pu concentration and the range of Pu-affinity for different sample types studied here and elsewhere^4,9,25,29^. The heterogeneous signal observed in bulk leach analysis of environmental samples demands further attention, with more focus on sophisticated particle characterization techniques.

**Figure 2:**
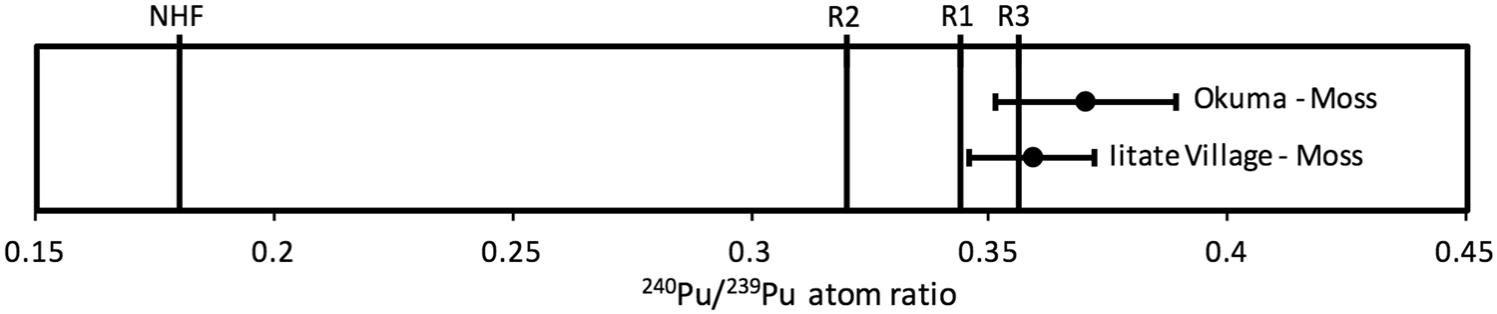
^240^Pu/^239^Pu atom ratios for moss samples from Okuma and Iitate village (2SE), FDNPP Reactor Units^27^ and Northern Hemisphere integrated global fallout (NHF).

Due to the limitations associated with bulk analysis, it is challenging to identify potential source terms that are end members of mixing combinations. For FDNPP-derived Pu in environmental materials, according to the ORIGEN estimated reactor values, the end-members of the ^240^Pu/^239^Pu mixing spectrum are global fallout (^240^Pu/^239^Pu atom ratio = 0.180 ± 0.014) and Reactor Unit 3 (^240^Pu/^239^Pu atom ratio = 0.356). It is not possible to identify Pu sourced from Reactor Units 1 (^240^Pu/^239^Pu atom ratio = 0.344) or 2 (^240^Pu/^239^Pu atom ratio = 0.320) because any ^240^Pu/^239^Pu atom ratios that correspond to these values would appear as a mixture of Pu from Reactor Unit 3 and global fallout^27,28,32^. Two moss samples collected from Iitate Village and Okuma exhibit ^240^Pu/^239^Pu atom ratios corresponding to Pu sourced from Reactor Unit 3 (Fig. 2) and are distinguishable from the average ORIGEN value of Reactor Unit 1^27^. This is the first report of Pu isotope ratios for environmental samples that correspond to a specific FDNPP reactor unit, but it is not possible to statistically evaluate this observation because no uncertainties are reported for the ORIGEN estimations^27^. There also remains some possibility that the ^240^Pu/^239^Pu atom ratios associated with these mosses may be a mixture of higher ^240^Pu/^239^Pu atom ratios from spent fuel ponds (SFPs) that have mixed with lower ^240^Pu/^239^Pu atom ratios such as global fallout. However, this scenario is unlikely considering SFP-derived Pu has not been observed in this work or in any other studies of FDNPP-derived Pu in the environment. Furthermore, it should be highlighted that both samples are mosses and highly unlikely to retain background Pu sourced from atmospheric fallout^4^.

A moss sample from Iitate Village exhibited the only ^240^Pu/^239^Pu atom ratio that did not align with either global fallout or the estimated ORIGEN model simulations (0.645 ± 0.045). A similar mean value was obtained for a vegetation sample collected from Odaka, Minamisoma (0.64 ± 0.37 (1σ)) by Schneider *et al*.^4^, but the relatively large uncertainty associated with this value indicates that it is not statistically different from the ORIGEN estimations. If material with this high ^240^Pu/^239^Pu atom ratio was sourced from FDNPP, it implies a higher fuel burn-up than otherwise estimated. A pressure-tube boiling-water reactor (RBMK) is the only reactor type reported in the literature that yields a ^240^Pu/^239^Pu atom ratio (0.67) corresponding to this anomalous value^33,34^. Extremely large quantities of radionuclides were released to the environment from a RBMK reactor during the Chernobyl accident in 1986. A recent study indicates that the more volatile Chernobyl-derived radionuclides contribute a significant propotion of background radiation levels in Japan^24^. However, ^240^Pu/^239^Pu atom ratios measured in soil samples collected after the Chernobyl accident, but before the FDNPP accident, were not indicative of Chernobyl-derived Pu^26^. Furthermore, ^240^Pu/^239^Pu atom ratios measured in environmental samples contaminated by the Chernobyl accident were found to be <0.60^35–38^. These observations infer that the anomalously high ^240^Pu/^239^Pu atom ratio measured here is unlikely to have been sourced from the Chernobyl disaster. Considering the complexity of the reactor conditions during the accident at FDNPP, and potential heterogeneity in ^240^Pu/^239^Pu atom ratios through partially-spent fuel, it is feasible that such conditions may have yielded a higher fuel burn-up. It should also be highlighted that the ^241^Pu/^239^Pu atom ratios observed elsewhere also point towards a high fuel burn-up^3,29,39^. Information regarding fuel burn-up can be related to accident mechanisms and is particularly valuable where high radiation levels prevent investigation of damaged reactors.

**Figure 3:**
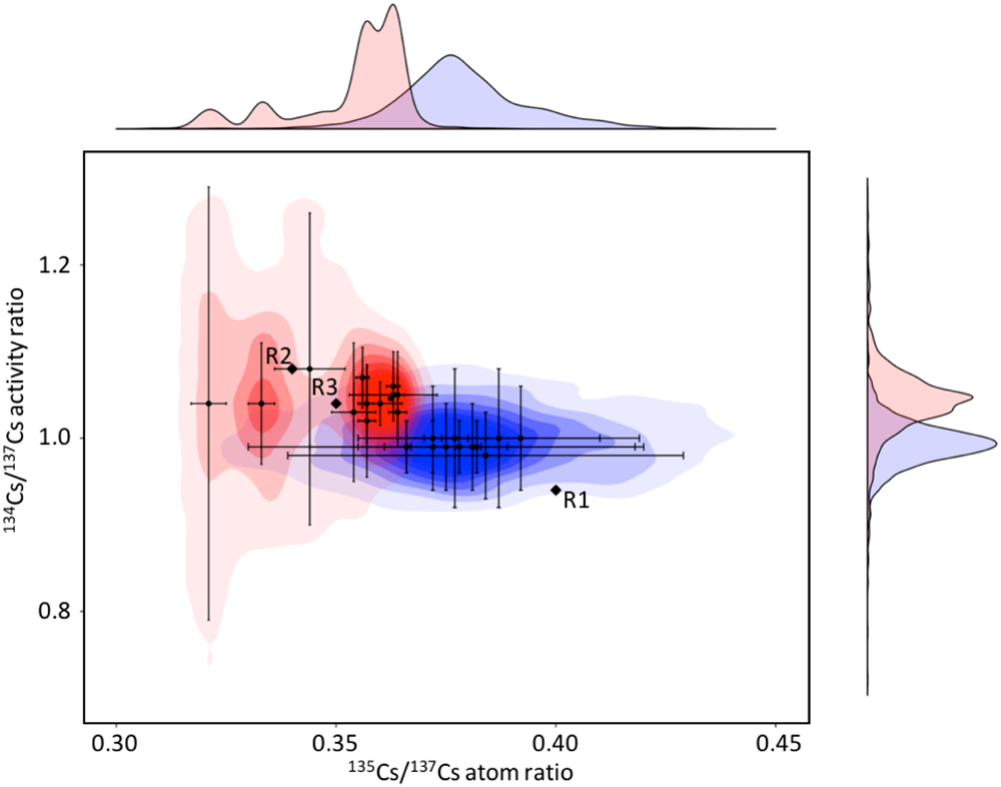
^135^Cs/^137^Cs atom ratios and ^134^Cs/^137^Cs activity ratios obtained in this study (samples collected northwest of FDNPP, red shading) and by Snow *et al*.^19^ (samples collected southwest of FDNPP, blue shading). Frequency distributions for ^135^Cs/^137^Cs atom ratios and ^134^Cs/^137^Cs activity ratios, respectively also illustrated. The larger diamond symbols correspond to the ORIGEN estimated reactor values^27^. Uncertainties are estimated to 2SE.

In order to discriminate potential source terms for the volatile Cs contamination, we supplement existing ^134,135^Cs/^137^Cs values and ^137^Cs specific activities for environmental samples from the region using TIMS and SF-ICPMS. Key to any interpretation of the Cs isoscape revealed in Fig. 1 is consideration of the competing influence of background signal prior to the accident and the extent of the mixing between this and one or more sources of Cs since the accident. Activity and isotopic composition before and after the event are required to deconvolve the source term and vector of transport. In addition to the isoscapes, we take advantage of a bivariate plot (Fig. 3) where ^134,135^Cs/^137^Cs data from this study are compared with data from Snow *et al*.^19^, which comprises 11 measurements of 5 environmental samples (1 soil, 4 vegetation) collected 100 to 250 km southwest of FDNPP^19^.

For sediments and vegetation samples obtained to the northwest of FDNPP, values for both ^134^Cs/^137^Cs and ^135^Cs/^137^Cs were obtained: 1.03–1.08 and 0.31–0.36, respectively. These data differ markedly from those established for sample material obtained from the southwest of FDNPP: 0.98–1.00 (^134^Cs/^137^Cs) and 0.37–0.39 (^135^Cs/^137^Cs) (Fig. 3)^19^. As mentioned previously, this could be a function of relative differences in global fallout contribution and/or mixing between one or more FDNPP source terms (fuel burn up and reactor conditions).

First, we consider the potential contribution of atmospheric fallout radiocaesium to the spatial pattern post-FDNPP. Atmospheric nuclear weapons tests conducted during the 1950s and 1960s and the Chernobyl disaster in 1986 have left a legacy of background radiocaesium throughout the Japanese terrestrial environment^24^. Remaining ^134^Cs (t_1/2_ = 2.07 a) from these releases is now negliglble, while ^137^Cs (t_1/2_ = 30.08 a) has decayed by a factor of <0.5 (Chernobyl) or <0.25 (atmospheric weapons testing) of the original concentrations. With a half-life of 2.3 × 10^6^ a, ^135^Cs totals remain effectively the same. In comparison to the releases from Reactor Units at FDNPP, these older releases had higher ^135^Cs/^137^Cs atom ratios (0.48–0.59 for Chernobyl fallout, 3.3–4.2 for atmospheric weapons testing; values decay corrected to 11/03/2011)^40^. A greater relative abundance of background radiocaesium will therefore increase ^135^Cs/^137^Cs atom ratios and decrease ^134^Cs/^137^Cs activity ratios, while larger fractions of FDNPP radiocaesium will have the opposite effect. The areas northwest of FDNPP received considerably more ^137^Cs contamination in comparison to the southwest^15^. The higher ^135^Cs/^137^Cs and lower ^134^Cs/^137^Cs in the southwest relative to the northwest may therefore be an indication of a relatively greater contribution from global fallout material.

Recently, Yang *et al*.^24^ analysed heavily-contaminated leaves to obtain isotope ratios for FDNPP radiocaesium that were expected to be unaffected by fallout. A mean ^134^Cs/^137^Cs activity ratio (1.033 ± 0.006) and ^135^Cs/^137^Cs atom ratio (0.334 ± 0.005) was recommended for all contamination from FDNPP. Subsequently, these values were used to estimate ^135^Cs activity in environmental samples prior to the FDNPP disaster^24^. For the data obtained in this study, the ^135^Cs/^137^Cs atom ratio for the highest activity vegetation sample (Katsurao moss = 1041 Bq g^−1^) differs from the Yang *et al*.^24^ recommended value by 6%. To attribute this variation solely to residual fallout material would require an inconceivable quantity of background radiocaesium. While we accept the quantity of fallout material may vary between different samples, it should be recognized that this may not be the only factor responsible for variation in ^134, 135^Cs/^137^Cs.

High activity samples from Katsurao and Minamisoma exhibit ^135^Cs/^137^Cs and ^134^Cs/^137^Cs are statistically indistinguishable (Table 1), as are samples from Okuma and Minamisoma. While it is possible that lower activity samples may have been shifted to equivalent residual fallout values, as discussed, it would require an inconceivable quantity of fallout ^135^Cs to do the same for higher activity samples. It is also highly unlikely that appropriate quantities of global fallout material have been retained in these environmental samples to coincidently yield equivalent ^135^Cs/^137^Cs and ^134^Cs/^137^Cs. We therefore conclude that these values are representative of FDNPP-derived radiocaesium and have not been significantly perturbed by global fallout material. Notably, these isotope ratios are in agreement with the ORIGEN estimated value for Reactor Unit 3. However, the radiocaesium isotope ratio values for Reactor Unit 3 fall in-between those of Reactor Units 1 and 2. Therefore, it is not possible to rule out a scenario where mixing between Reactor Unit 1 and 2 generated these isotope ratios. Furthermore, as is the case for Pu, the Cs isotope ORIGEN values should be used with some caution given that they correspond to a mean value with no reported uncertainty. It is also interesting to note that the difference between the signature proposed here and that presented in Yang *et al*.^24^ occurs even though the samples from the respective studies were collected from locations within relatively close proximity to one another: the highest activity samples measured in Yang *et al*.^24^ were collected from Namie Town and Minamisoma, while the highest activity samples in this work were collected from Minamisoma and Katsurao. This indicates variability in the isotopic composition of radiocaesium in a more localized manner than the proposed northwest-southwest trend. Further work is required to explore this further, ideally on material that does not contain fallout material such as isolated FDNPP particles.

**Table 1:** Radiocaesium isotopic data for samples that exhibit consistent isotopic ratios with Reactor Unit 3. Uncertainties estimated to 2SE.

Sample location	Sample type	^134^Cs/^137^Cs activity ratio	^135^Cs/^137^Cs atom ratio	^137^Cs specific activity* (Bq g^−1^)
Minamisoma	Road side dust	1.02 ± 0.07	0.357 ± 0.002	114 ± 9
Minamisoma	Sediment	1.04 ± 0.05	0.357 ± 0.002	778 ± 58
Katsurao	Moss	1.07 ± 0.04	0.356 ± 0.002	1040 ± 77
Okuma	Soil	1.03 ± 0.08	0.354 ± 0.005	53.12 ± 4
Reactor Unit 3 (ORIGEN)		1.04	0.356	—

*Activity per unit mass of leached (8 M HNO_3_) sub-sample (~1 g).

The issue of global fallout inhibiting interpretation of isotopic ratios in environmental samples highlights the requirement for more sophisticated particle characterisation techniques. For radiocaesium, we recommend that isotopic analysis should be performed on a suite of FDNPP fallout material ranging from sub-millimeter heterogenous agglomerations^41^ to smaller micron-sized spherical Cs-rich particles^42,43^. Analysis of material collected on atmospheric sampling filters is also expected to provide Cs isotope ratios that are not influenced by global fallout material.

In addition to the effects of global fallout material, mixing between FDNPP source terms is also expected to be a cause of spatial trends in the isotopic composition of radiocaesium. The contributing factors for this are a function of several variables:

- Distinctive ^134^Cs/^137^Cs and ^135^Cs/^137^Cs associated with each of the FDNPP Reactor Units 1–3^27,32^. In addition, ^135^Cs/^137^Cs atom ratios are expected to vary through reactor cores, therefore individual reactor cores may have emitted inconsistent ^135^Cs/^137^Cs^44^.
- The Reactor Units released material in variable quantities between 12/03/2011 and 23/03/2011^1^: For total ^137^Cs release by FDNPP, reported contributions for Reactor Units 1, 2 and 3 are 3.18%, 91.5% and 4.64%, respectively^24^.
- Upon release, transportation of volatile radionuclides such as radiocaesium was defined by wind direction, while the predominant cause of deposition was precipitation^1^.

It is the interaction between these processes that governed spatial distribution of radiocaesium isotopes in the environment: The location of radiocaesium contamination in the environment with a specific isotopic signature will reflect the proportional amounts of material emitted from the respective reactors, their corresponding isotopic composition, the wind direction that coincides with the time of release and the location of the plume when rainout occurred. The location of rainout is reported to be the cause of highest radiocaesium deposition^1,45,46^. There were two plumes released from FDNPP that were affected by precipitation:

1. An explosion in Reactor Unit 2 on the morning of 15/03/2011 released a plume of contamination that was subsequently transported north-northwest by prevailing wind. Rainout occurred during the afternoon of 15/03/2011 and continued until the morning of 16/03/2011^1,23^. These events are acknowledged to be the cause of the high ^137^Cs activities monitored to the northwest of FDNPP^19^.
2. On 20/03/2011 a release from Reactor Unit 1 was transported south with a plume previously emitted from Reactor Unit 1 (Plume 1) on 12/03/2011. Prevailing winds carried the contamination southwards to the Tokyo Metropolitan Area when precipitation occurred^1^.

The contamination associated with these deposition events is a mixture of radiocaesium sourced from Reactor Units 1, 2 and 3. However, according to information obtained from air pollution monitoring stations it is apparent that the material in the northwest is predominantly sourced from Reactor Unit 2, while the radiocaesium deposited in the south is reported to consist of a comparatively larger fraction of material from Reactor Unit 1^1^. We see that this is reflected in the radiocaesium isotope ratio data: samples collected northwest of FDNPP ^134^Cs/^137^Cs and ^135^Cs/^137^Cs are weighted towards Reactor Units 2 and 3 and samples from south of FDNPP appear to contain more radiocaesium from Reactor Unit 1 (Fig. 3). Contrary to these trends however, are environmental samples from Tsukuba (^135^Cs/^137^Cs atom ratio = 0.341 ± 0.016) and Kamagaya (^135^Cs/^137^Cs atom ratio = 0.355 ± 0.0020), located 140 km and 230 km southwest of FDNPP respectively^47^. These values are in closer agreement with the samples collected northwest of FDNPP in this study than those from the southwest^19^. Considering Reactor Units 1, 2 and 3 all contributed to multiple plumes that may have undergone multiple release events^1^, one might expect to observe this wider spectrum of Cs isotope ratio values. Such observations again highlight the requirement for a larger data set with respect to ^134, 135^Cs/^137^Cs particularly for southwest of FDNPP and provide yet another example of the complexity of the FDNPP Cs isoscape.

It is evident that both global fallout and the interplay between reactor release chronology and meteorological conditions have contributed to the observed spatial trends in isotopic signature of radiocaesium. The effect of global fallout has previously been confirmed and is certainly responsible for some of the variation in ^134,135^Cs/^137^Cs^24^. However, the work conducted here, combined with data and insights from elsewhere, indicates that the influence of reactor emission chronology in combination with prevailing meteorological conditions is the more dominant factor responsible for the lower ^135^Cs/^137^Cs and higher ^134^Cs/^137^Cs in the northwest relative to southwest FDNPP^6,17–19,24^. Isotopic analyses of isolated radiocaesium-rich particulates in environmental materials and air filters that captured FDNPP contamination during different periods is required to improve our understanding of these processes. Constraining the spatial distribution of the isotopic signature of radiocaesium facilitates the tracing of post-depositional migration of these radionuclides through the environment, which is key for remediation efforts and public health. The use of ^134^Cs/^137^Cs activity ratios in combination with the more sensitive ^135^Cs/^137^Cs atom ratios demonstrates how these techniques may be used to explore the transport and behavior of radiocaesium through the environment.

## Methods

### Sampling

A range of environmental samples (soil, sediment and various types of vegetation), were collected from several locations in the Fukushima prefecture, the mouth of the Abukuma River and one sample from the Chiba prefecture during 2014 and 2015. A joint sampling effort was conducted by the University of Bristol and Kyoto University. Sample descriptions are available in supplementary information (Table S1).

### Analytical procedures

A portion of dried sample (~1 g) was dry-ashed at 450 °C for 12 hours before being transferred to a polytetrafluoroethylene vial and spiked with ~1 mBq ^242^Pu (IRMM-085, Certified Spike Isotopic Reference Material, Institute of Reference Materials and Methods, Brussels, Belgium). Samples were refluxed overnight at 165 °C using 8 M HNO_3_ (all reagents used were analytical grade or higher purity). Particulates were separated from the leachate by centrifugation at 4000 rpm for 10 minutes. Supernatant was dried down and re-dissolved in 10 ml 8 M HNO_3_ − 0.2 M NaNO_2_ and heated at 90 °C for 20 minutes. BioRad AG1-X8 Cl^−^ form anion exchange resin (~1 ml) was placed into a column with dimensions of 30 × 80 mm and pre-conditioned with 10 ml 8 M HNO_3_. Sample was loaded onto the column in two 5 ml aliquots, the two aliquots, containing species with no affinity for the resin, were collected and kept for Cs separation. The columns were subsequently rinsed with 10 ml 3 M HNO_3_, 10 ml 9 M HCl and eluted with 12 ml 9 M HCl-0.1 M NH_4_I. The eluent was gently dried down and the precipitate was oxidised by refluxing with 1 ml concentrated (69% v/v) HNO_3_ and 1 ml concentrated H_2_O_2_ (30% v/v) overnight at 165 °C. The sample was adjusted to 2 ml 0.6 M HNO_3_ for analysis by MC-ICPMS.

Plutonium isotopic measurements were conducted using a Neptune multi-collector inductively coupled plasma mass spectrometer (MC-ICPMS) (Thermo Fisher Scientific Inc., MA, USA), at the Bristol Isotope Group, University of Bristol. An Aridus sample introduction system (CETAC Technologies Inc., NE, USA) was connected to a microflow PFA-50 nebuliser for sample uptake. The instrument was tuned daily using a 30 ppb natural U standard (CRM U112a). All analyses were performed in low-resolution mode (m/Δm ≈ 400). Plutonium isotopes (^239, 240, 242^Pu) were measured via peak-jumping mode using the axial secondary electron multiplier (SEM). ^238^U^+^ was simultaneously monitored by Faraday cups L1, L2, L3 (Table S3). SEM dark noise remained at <3 cpm. The SEM sits behind a retarding potential quadrupole lens (RPQ), which is a highly selective filter that removes ions with a disturbed energy or trajectory improving abundance sensitivity. In the case of Pu, the RPQ is particularly advantageous for reducing tailing effects from large ^238^U^+^ beams on ^239^Pu.

Uranium decontamination factors for the chemical separation procedure for certified reference material IAEA-385 (estuarine sediment) were 1.5 × 10^3^ (n = 5). The procedure gave a chemical yield of approximately 80%. For Pu measurements by MC-ICPMS, residual ^238^U may form a hydride (^238^UH^+^) in the interface region of the instrument and interfere at *m/z* 239. Tailing effects from ^238^U^+^ may also interfere at *m/z* 239. To account for this, a solution of CRM U112a yielding 8 V (≈5 × 10^8^ cps) was monitored periodically during each measurement sequence. (^238^UH^+^  + U_tail_)/^238^U^+^ was typically 1 × 10^−6^ and the ^238^U^+^ intensity for the FDNPP Pu samples ranged from 100–300 mV. Repeat measurements of CRM U112a were also performed throughout the measurement sequence to correct for instrumental mass bias.

Repeat measurements of certified reference materials IAEA-367, IAEA-384 and IAEA-385 validate the accuracy and precision of this methodology for the determination of ^239+240^Pu activity and ^240^Pu/^239^Pu atom ratios in complex environmental materials (Tables S4–S7). Sub-samples of IAEA-385 were processed alongside FDNPP samples to ensure analytical proficiency was upheld.

Caesium was successfully separated from the retained solution using a double cation exchange method with ammonium molybdophosphate-polyacrylonitrile (AMP-PAN). Purified Cs was loaded onto a single standard-purity Re filament with glucose activator for analysis by TIMS. This method is described in Dunne *et al*.^20^.

To determine ^137^Cs activity of the leachate, ^137^Cs/^133^Cs atom ratios measured by TIMS were combined with ^133^Cs concentration measurements of a fraction of the initial leachate. The ^133^Cs concentration was determined using a Thermo Element 2XR sector field-inductively coupled plasma mass spectrometer (SF-ICPMS). All samples were introduced in 2 ml 0.6 M HNO_3_ using a PFA nebuliser (ESI Scientific, Omaha, NE) and a quartz spray chamber introduction system. Measurements were performed in low resolution mode as there are no significant interferences at *m/z* 133. Analytical grade ^133^Cs standards (1 g ml^−1^) were diluted accordingly and used as concentration standards (Sigma-Aldrich, Steinheim, Germany). Concentrations are derived from calibration curves (n = 5), spanning the range of beam intensities produced by the respective samples (Table S8).

## Electronic supplementary material

Supplementary Information

## Data Availability

All data generated in this study are included in Supplementary Information.
