# Supplementary material for: Spatial pattern of plutonium and radiocaesium contamination released during the Fukushima Daiichi nuclear power plant disaster
Source: Sci Rep. 2018 Nov 14;8:16799. doi: 10.1038/s41598-018-34302-0 (PMC6235829; doi:10.1038/s41598-018-34302-0)
Supplement: Supplementary file 1 — Supplementary Information [file 41598_2018_34302_MOESM1_ESM.docx]

Supplementary Information

**Spatial pattern of plutonium and radiocaesium contamination released during the Fukushima Daiichi nuclear power plant disaster**

James A. Dunne^1,2^ *, Peter G. Martin^3^, Yosuke Yamashiki^4^, Ian X. Y. Ang^3^, Tom B. Scott^3^, David A. Richards^1,2^ *

^1^ School of Geographical Sciences, University of Bristol, University Road, Bristol BS8 1SS, UK

^2^ Bristol Isotope Group, University of Bristol, Wills Memorial Building, Queen's Road

Bristol BS8 1RJ

^3^  Interface Analysis Centre, HH Wills Physics Laboratory, University of Bristol, Bristol BS8 1TL, UK

^4^ Graduate School of Advanced Integrated Studies in Human Survivability, Kyoto University, Kyoto 606-8501, Japan

* Author for correspondence: james.dunne@bristol.ac.uk, david.richards@bristol.ac.uk

**Table S1:** Description and location of environmental samples analysed in this study. All materials were collected from the surface.

| **Sample-ID** | **Location description** | **Material description** | **Classification for**  **Fig. 1** | **Latitude (^o^N)** | **Longitude**  **(^o^E)** |
| --- | --- | --- | --- | --- | --- |
| MSO1-CS | Minamisoma | Coarse sediment | Sediment | 37.69556 | 140.90750 |
| MSO1-RSD | Minamisoma | Road side dust | Sediment | 37.68056 | 140.89250 |
| MSO2 | Minamisoma | Bark/leaf litter | Vegetation | 37.58528 | 140.88920 |
| MSO3 | Minamisoma | Soil | Sediment | 37.60639 | 140.93028 |
| MSO4 | Minamisoma | Sediment | Sediment | 37.58861 | 140.89167 |
| IT1-FS | Iitate | Fine sediment | Sediment | 37.61417 | 140.70778 |
| IT1-MS | Iitate | Moss and soil mix | Vegetation | 37.60417 | 140.71280 |
| IT1-M | Iitate | Moss | Vegetation | 37.61416 | 140.70778 |
| IT1-RSD | Iitate | Road side dust | Sediment | 37.61417 | 140.70780 |
| ITV | Iitate Village | Moss | Vegetation | 37.66255 | 140.77456 |
| KTS1 | Katsurao hillside | Soil | Sediment | 37.50750 | 140.83167 |
| KTS2 | Katsurao track | Moss and soil mix | Vegetation | 37.50667 | 140.83389 |
| ABK1 | Abukuma river | Sediment | Sediment | 38.06250 | 140.90722 |
| ABK2 | Abukuma river | Sediment | Sediment | 38.06250 | 140.90722 |
| DT1 | Date | Mud | Sediment | 37.79916 | 140.61750 |
| DT2 | Date | Mud | Sediment | 37.81167 | 140.61750 |
| CHB | Chiba, Tokyo | Soil | Sediment | 35.60667 | 140.18472 |
| RZ1-S | Nagadoro | Sediment | Sediment | 37.60944 | 140.74167 |
| RZ1-M | Nagadoro | Moss | Vegetation | 37.60944 | 140.74167 |
| MSA-1 | Minamiosawa | Moss, leaf litter | Vegetation | 37.54138 | 140.42333 |
| OKU-S | Okuma | Soil | Sediment | 37.41000 | 140.97944 |
| OKU-MS | Okuma | Moss and soil mix | Vegetation | 37.41000 | 140.97944 |

**Table S2:** Pu and Cs isotope ratios and specific activity (strong acid leach fraction of ~ 1g sub-samples) for environmental samples analysed in this study. Also included is specific activity for total sample (^137^Cs). Uncertainties for the mass spectrometry measurements are estimated to 2SE. Bulk gamma spectrometry measurements are reported as standard deviation with a coverage factor of 2.

**Table S3:** Cup configuration used for plutonium isotopic measurements by MC-ICP-MS.

| **L3** | **L2** | **L1** | **Axial SEM** |
| --- | --- | --- | --- |
|  |  | ^238^U^+^ | ^239^Pu^+^ |
|  | ^238^U^+^ |  | ^240^Pu^+^ |
| ^238^U^+^ |  |  | ^242^Pu^+^ |

**Table S4:** Repeat measurements of the same separated IAEA-367 solution over several weeks. Uncertainties on individual measurement points estimated as standard error with a coverage factor of 2.

| **Study** | **^240^Pu/^239^Pu atom ratio** |
| --- | --- |
| This Study | 0.289 ± 0.001 |
|  | 0.291 ± 0.001 |
|  | 0.290 ± 0.003 |
|  | 0.289 ± 0.002 |
|  | 0.294 ± 0.002 |
|  | 0.293 ± 0.002 |
|  | 0.290 ± 0.003 |
| **Mean (n=7)** | **0.290 ± 0.002 (external standard deviation)** |
| Kim et al. (2000) | 0.30 ± 0.03 (one standard deviation) |
| Muramatsu et al. (2001) | 0.303 ± 0.007 (external standard deviation n = 4) |
| Lindahl et al. (2010) | 0.297 - 0.310 |
| Lindahl et al. (2011) | 0.293 ± 0.002 (standard uncertainty n = 6) |
| Lindahl et al. (2012) | 0.297 ± 0.008 (standard uncertainty n = 10) |
| Xu et al. (2014) | 0.296 ± 0.008, 0.290 ± 0.009 (expanded uncertainty k = 1) |

**Table S5:** Repeat measurements of the same separated IAEA-384 solution over several weeks. Uncertainties on individual measurement points estimated as standard error with a coverage factor of 2.

| **Study** | **^240^Pu/^239^Pu atom ratio** |
| --- | --- |
| This Study | 0.0511 ± 0.0002 |
|  | 0.0511 ± 0.0001 |
|  | 0.0508 ± 0.0003 |
|  | 0.0510 ± 0.0003 |
|  | 0.0515 ± 0.0003 |
|  | 0.0518 ± 0.0004 |
|  | 0.0515 ± 0.0004 |
| **Mean** | **0.0513 ± 0.0003 (external standard deviation n = 7)** |
| Lee et al. (2001) | 0.0483 ± 0.048 (external standard deviation n = 4) |
| Lindahl et al. (2011) | 0.049 ± 0.001 (standard uncertainty n = 6) |
| Povinec et al. (2007) | 0.049 ± 0.001 (external standard deviation n = 9) |

**Table S6:** Repeat ^240^Pu/^239^Pu atom ratio measurements of separate sub-samples of IAEA-385 independently prepared and analysed. Uncertainties on individual measurement points estimated as standard error with a coverage factor of 2.

| **Study** | **^240^Pu/^239^Pu atom ratio** |
| --- | --- |
| This Study | 0.178 ± 0.007 |
|  | 0.179 ± 0.007 |
|  | 0.191 ± 0.006 |
|  | 0.179 ± 0.006 |
|  | 0.181 ± 0.003 |
|  | 0.184 ± 0.005 |
|  | 0.176 ± 0.004 |
|  | 0.178 ± 0.002 |
|  | 0.178 ± 0.002 |
| **Mean (n=9)** | **0.181 ± 0.005 (external standard deviation)** |
| Cizdziel et al. (2008) | 0.193 ± 0.003, 0.192 ± 0.004 (one standard deviation) |
| Lindahl et al. (2010) | 0.188 ± 0.007 (standard uncertainty) |
| Lindahl et al. (2012) | 0.179 ± 0.011 (standard uncertainty n = 9) |
| Pham et al. (2008) | 0.174 ± 0.016 (one standard deviation) |

**Table S7:** Repeat ^239+240^Pu activity measurements of separate sub-samples of IAEA-385 independently prepared and analysed. Uncertainties on individual measurement points estimated as standard error with a coverage factor of 2.

| **Study** | **^239+240^Pu activity (Bg kg^-1^)** |
| --- | --- |
| This Study | 2.56 ± 0.21 |
|  | 2.89 ± 0.18 |
|  | 2.86 ± 0.18 |
|  | 2.69 ± 0.15 |
|  | 2.82 ± 0.45 |
|  | 2.74 ± 0.31 |
| **Mean (n=6)** | **2.76 ± 0.12 (external standard deviation, n = 7)** |
| Varga et al. (2007) | 2.75 ± 0.15 (one standard deviation) |
| Cizdziel et al. (2008) | 3.10 ± 0.04, 3.03 ± 0.03 (one standard deviation) |
| Qiao et al. (2011) | 2.98 ± 0.08 (one standard deviation) |
| Lindahl et al. (2012) | 2.58 ± 0.04 (standard uncertainty) |
| Pham et al. (2008) | 2.94 ± 0.09 (one standard deviation) |

**Table S8:** Repeat ^137^Cs activity measurements of separate sub-samples of IAEA-330 independently prepared and analysed. Values have been decay corrected to the certificate value, 15/10/2007. Uncertainties on individual measurement points estimated as standard deviation with a coverage factor of 2.

| **Study** | **^137^Cs activity (Bg g^-1^)** |
| --- | --- |
| This study | 1.20 ± 0.08 |
|  | 1.17 ± 0.07 |
|  | 1.31 ± 0.11 |
| **Mean (n=3)** | **1.22 ± 0.07 (external standard deviation)** |
| IAEA certified value | 1.24 ± 0.04 (95 % confidence level) |
